# LncRNA *MALAT1* enhances oncogenic activities of EZH2 in castration-resistant prostate cancer

**DOI:** 10.18632/oncotarget.5728

**Published:** 2015-10-15

**Authors:** Dejie Wang, Liya Ding, Liguo Wang, Yu Zhao, Zhifu Sun, R. Jeffrey Karnes, Jun Zhang, Haojie Huang

**Affiliations:** ^1^ Department of Biochemistry and Molecular Biology, Mayo Clinic College of Medicine, Rochester, MN 55905, USA; ^2^ Department of Urology, Mayo Clinic College of Medicine, Rochester, MN 55905, USA; ^3^ Mayo Clinic Cancer Center, Mayo Clinic College of Medicine, Rochester, MN 55905, USA; ^4^ Department of Medical Statistics and Informatics, Mayo Clinic College of Medicine, Rochester, MN 55905, USA; ^5^ Department of Laboratory Medicine and Pathology, Mayo Clinic College of Medicine, Rochester, MN 55905, USA

**Keywords:** MALAT1, EZH2, castration-resistant prostate cancer (CRPC), Polycomb repressive complex 2 (PRC2)

## Abstract

The Polycomb protein enhancer of zeste homolog 2 (EZH2) is frequently overexpressed in advanced human prostate cancer (PCa), especially in lethal castration-resistant prostate cancer (CRPC). However, the signaling pathways that regulate EZH2 functions in PCa remain incompletely defined. Using EZH2 antibody-based RNA immunoprecipitation-coupled high throughput sequencing (RIP-seq), we demonstrated that EZH2 binds to *MALAT1*, a long non-coding RNA (lncRNA) that is overexpressed during PCa progression. GST pull-down and RIP assays demonstrated that the 3′ end of *MALAT1* interacts with the N-terminal of EZH2. Knockdown of *MALAT1* impaired EZH2 recruitment to its target loci and upregulated expression of EZH2 repressed genes. Further studies indicated that *MALAT1* plays a vital role in EZH2-enhanced migration and invasion in CRPC cell lines. Meta-analysis and RT-qPCR of patient specimens demonstrated a positive correlation between *MALAT1* and EZH2 expression in human CRPC tissues. Finally, we showed that *MALAT1* enhances expression of PRC2-independent target genes of EZH2 in CRPC cells in culture and patient-derived xenografts. Together, these data indicate that *MALAT1* may be a crucial RNA cofactor of EZH2 and that the EZH2-*MALAT1* association may provide a new avenue for development new strategies for treatment of CRPC.

## INTRODUCTION

Prostate cancer (PCa) is the most commonly diagnosed malignancy and the second leading cause of cancer death among American men. Androgen deprivation therapy (ADT) is the mainstay of treatment for advanced/metastatic PCa. However, a significant portion of patients experience disease relapse and tumors eventually evolve into castration resistant prostate cancer (CRPC), which is the leading cause of PCa death at present [1].

LncRNAs have been implicated in PCa development and progression [2]. Metastasis-associated lung adenocarcinoma transcript-1 (*MALAT1*) lncRNA was originally identified as a prognostic factor of tumor development and metastasis in various types of human tumors such as glioma, lung, pancreatic and prostate cancer as well as esophageal squamous cell carcinoma [3–6]. *MALAT1* was found positively correlated with Gleason score, the level of prostate specific antigen (PSA), tumor stage and castration resistance in PCa [7]. *MALAT1* overexpression appears to be a promising diagnostic urinary biomarker for prostate cancer [8]. Moreover, *MALAT1* was identified as the most highly expressed transcript in CRPC by whole transcriptome sequencing in a panel of CRPC bone marrow biopsy specimens [9]. However, the role of *MALAT1* in development and progression in PCa, especially CRPC remains elusive.

EZH2, working with EED and SUZ12, the other two essential components of the Polycomb repressive complex-2 (PRC2), functions primarily as a methyltransferase catalyzing histone H3 lysine 27 trimethylation (H3K27me3) and promoting gene silencing [10]. EZH2 has been found frequently overexpressed in variety of human cancers such as prostate and breast cancer [11, 12]. Increasing evidences show that EZH2 levels correlate with increased proliferation rates, invasiveness and metastasis of PCa in patients [13, 14]. Moreover, it has been shown that EZH2 interacts with the *HOTAIR* lncRNA and facilitates PRC2 targeting in the genome of breast cancer and promotes breast cancer metastasis [15]. Further studies reveal that the EZH2-*HOTAIR* interaction is regulated by various signaling pathways such as cyclin-dependent kinases (CDKs) and the tumor suppressor protein BRCA1 [16, 17]. *PCAT1* was identified as a prostate-specific lncRNA that can bind to EZH2, but the expression of *PCAT-1* and EZH2 is nearly mutually exclusive in human PCa [2]. It thus remains unclear which lncRNAs are required for EZH2 functions to facilitate PCa progression.

Despite the fact that *MALAT1* is often overexpressed in human cancers including PCa, its functional role in cancer progression is poorly understood. One study demonstrates previously that *MALAT1* associates with PRC2 by interacting with SUZ12 but not EZH2 and that inhibition of *MALAT1* decreases the binding of SUZ12 to the E-cadherin gene promoter in bladder cancer [18]. In contrast, a recent study reports that *MALAT1* can bind to EZH2 and downregulate E-cadherin expression through EZH2-mediated H3K27me3 at the E-cadherin gene promoter in clear renal cancer [19]. Given that the previous findings regarding the mechanism by which *MALAT1* affects the function of PRC2 are not consistent, further investigation is warranted. In the present study, we identified *MALAT1* as a vital regulator of EZH2 in CRPC cells. We further showed that *MALAT1* interacts with and facilitates EZH2 occupancy and the H3K27me3 activity of EZH2 in CRPC cells and that expression of *MALAT1* correlates with EZH2 levels in human CRPC specimens.

## RESULTS

### Identification of *MALAT1* as an EZH2-binding lncRNA by RIP-seq in PCa cells

**Figure 1:**
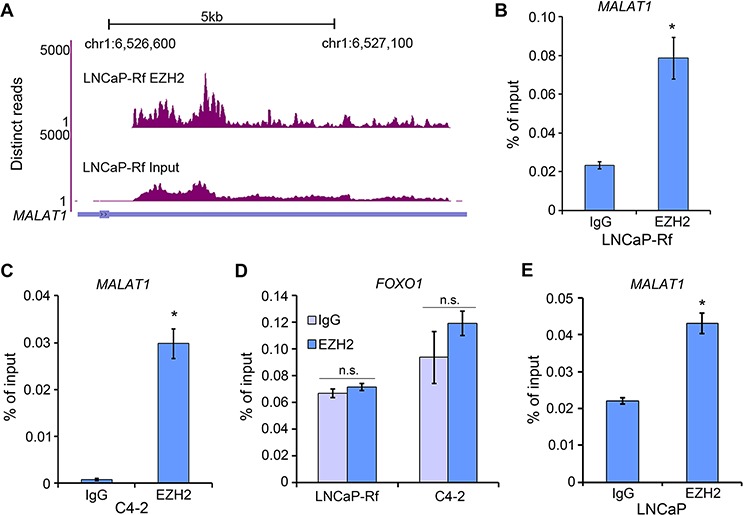
*MALAT1* binds to EZH2 in PCa cells **A.** RIP-seq profile showing the pulldown of *MALAT1* by EZH2. Screen shots of the UCSC genome browser showing coverage profiles of *MALAT1* in EZH2 RIP-seq and input samples. Both RIP-seq and input samples were normalized into the same sequencing depth. **B–D.** LNCaP-Rf and C4-2 CRPC cells were harvested for RIP with anti-EZH2 antibody or control IgG. Bound *MALAT1* (B, C) and *FOXO1* mRNA (negative control) (D) were analyzed by RT-qPCR (*n* = 3). Data are means ± S.D. from experiments with three replicates. *P < 0.01 comparing to the IgG control group. n.s., not statistically significant. **E.** RT-qPCR analysis of *MALAT1* in RIP samples pulled down by anti-EZH2 antibody in LNCaP androgen-sensitive cells. *P < 0.01 comparing to the IgG control group.

Previous studies show that lncRNAs such as *HOTAIR* play important roles in facilitating genome-wide occupancy of EZH2 onto chromatin in breast cancer cells [15]. However, *HOTAIR* is hardly detected in human PCa specimens [2], suggesting that other lncRNAs may be important for EZH2 function in PCa. To identify EZH2 interacting lncRNA(s) in PCa cells, unbiased RNA immunoprecipitation (RIP)-coupled high throughput sequencing (RIP-seq) was used. Given that the crosslink-based RIP is highly susceptible to RNA contamination [20], we performed native (without crosslink) EZH2 RIP-seq in LNCaP-Rf CRPC cells [21]. *MALAT1* was identified as one of the lncRNAs that bind to EZH2 (Figure 1A). The binding of *MALAT1* with EZH2 was further confirmed by RIP-qPCR in LNCaP-Rf and C4-2, another CRPC cell line (Figure 1B and 1C). No EZH2 binding to other RNA species such as *FOXO1* mRNA was detected in both cell lines (Figure 1D). These data suggest that EZH2 specifically binds to *MALAT1* in CRPC cells. RIP-qPCR analysis showed that *MALAT1* bound to EZH2 in androgen-responsive cell line LNCaP (Figure 1E), suggesting that *MALAT1* also impacts EZH2 functions in androgen-sensitive PCa cells.

### Identification of region(s) in *MALAT1* and EZH2 responsible for their interaction

**Figure 2:**
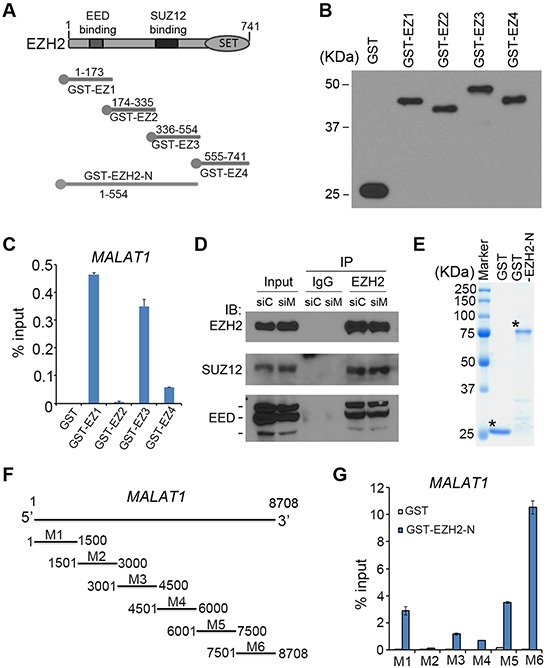
Analysis of the regions in *MALAT1* and EZH2 responsible for their interaction **A, B.** Schematic diagram and western blot detection of GST and GST-EZH2 recombinant proteins indicated. **C.** RT-qPCR analysis of *MALAT1* lncRNA pulled down by GST or GST-EZH2 recombinant proteins. Data are means ± S.D. from experiments with three replicates. **D.** C4-2 cells were transfected with non-specific control (siC) or a pool of *MALAT1*-specific siRNAs (siM) for 48 h and cells were harvested for immunoprecipitation using control IgG or EZH2 antibody and western blot using indicated antibodies. **E.** Coomassie blue staining of GST and GST-EZH2-N (amino acids 1–554) fusion protein. Asterisks indicate the proteins with correct molecular mass. **F.** Schematic diagram of *MALAT1* regions M1-M6 used for GST pull down assays. **G.** RT-qPCR analysis of the *in vitro* transcribed different regions of *MALAT1* lncRNA pulled down by GST or GST-EZH2-N (1–554) recombinant proteins. Data are means ± S.D. from experiments with three replicates.

To determine which region(s) of EZH2 protein mediate its interaction with *MALAT1*, we performed glutathione S-transferase (GST) pulldown assays. GST-EZH2 recombinant proteins were purified as we described previously [17] and GST pull-down assays were performed using C4-2 cell lysates (Figure 2A and 2B). We found that *MALAT1* preferentially bound to EZH2 in two regions (amino acids 1–173 and 336–554), which are known to bind to EED and SUZ12, respectively (Figure 2B and 2C). It is worth noting that knockdown of *MALAT1* had no effect on EZH2 binding with EED and SUZ12 in C4-2 cells (Figure 2D). Additionally, we performed reciprocal *in vitro* RNA binding assays. *In vitro* transcribed *MALAT1* fragments (M1-M6) were incubated with GST-EZH2-N (amino acids 1–554), the region that strongly binds to *MALAT1*, and RT-qPCR was used to detect the RNAs pulled down by GST-EZH2-N. We demonstrated that the region containing the nucleotides 7501–8708 at the 3′ end of *MALAT1* interacted strongly with EZH2-N (Figure 2E–2G). These data indicate that *MALAT1* directly interacts with the N-terminal of EZH2 and EZH2 interacts with the 3′ end of *MALAT1*, at least under *in vitro* conditions.

### *MALAT1* is important for EZH2-mediated silencing of Polycomb-dependent target genes

**Figure 3:**
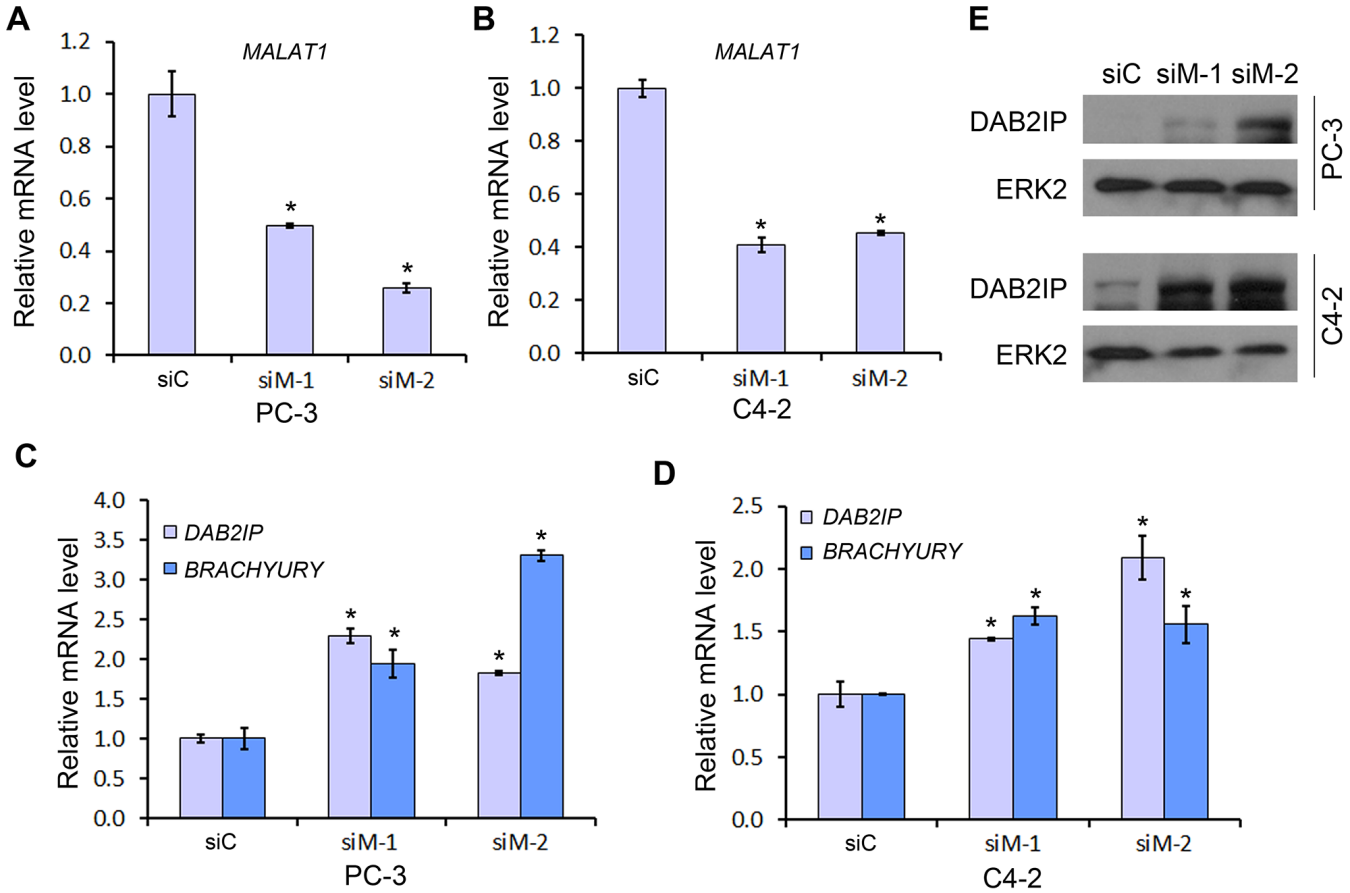
The effect of *MALAT1* on expression of Polycomb-dependent target genes of EZH2 **A–D.** PC-3 (A, C) and C4-2 (B, D) cells were transfected with non-specific control (siC) or two individual *MALAT1*-specific siRNAs (siM-1 and siM-2) for 48 h and then cells were harvested for RT-qPCR analysis of *MALAT1* (A, B) and EZH2 Polycomb-dependent target genes *DAB2IP* and *BRACHYURY* (C, D). Data are means ± S.D. from experiments with three replicates. *P < 0.01 comparing to the siC group. **E.** PC-3 and C4-2 cells were transfected as in (A) and expression of DAB2IP proteins were analyzed by western blot. ERK2 was used as a loading control.

EZH2 is a core subunit of PRC2 that promotes gene silencing via its histone methyltransferase activity. To investigate the role of *MALAT1* on EZH2-mediated Polycomb-dependent gene silencing, we employed two independent *MALAT1* siRNA (siM-1 and siM-2) to knock down *MALAT1* in PC-3 and C4-2 CRPC cell lines. We demonstrated that expression of *MALAT1* was effectively knocked down by *MALAT1* siRNAs in both cell lines (Figure 3A and 3B). Importantly, *MALAT1* knockdown by both siRNAs invariably resulted in derepression of EZH2 repressed genes examined including *DAB2IP* and *BRACHYURY* (or called *T* gene) (Figure 3C and 3D). Western blot analysis showed that *MALAT1* knockdown also increased DAB2IP protein levels in both PC-3 and C4-2 cell lines (Figure 3E). These results suggest that *MALAT1* enhances EZH2-mediated repression of Polycomb-dependent target genes in CRPC cells.

### *MALAT1* is important for EZH2 occupancy and H3K27me3 levels at Polycomb target loci

**Figure 4:**
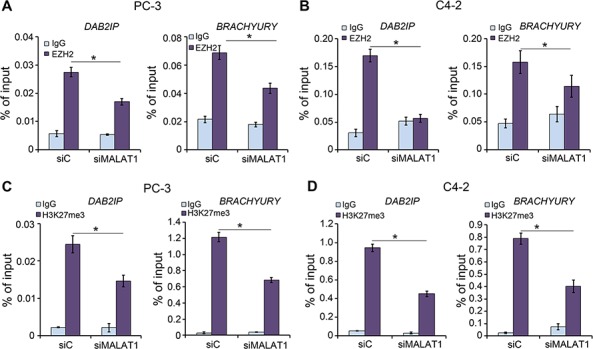
The effect of *MALAT1* on EZH2 recruitment and H3K27me3 levels at EZH2 Polycomb-dependent target gene loci **A, B.** ChIP-qPCR analysis of EZH2 occupancy at its target loci *DAB2IP* and *BRACHYURY*. PC-3 (A) and C4-2 (B) cells were transfected with non-specific control (siC) or a pool of *MALAT1*-specific siRNAs (siMALAT1). 48 h after transfection, cells were harvested for ChIP assay with non-specific IgG or EZH2 antibody. Data are means ± S.D. from experiments with three replicates. *P < 0.01. **C, D.** ChIP-qPCR analysis of H3K27me3 levels at the promoters of EZH2 target loci *DAB2IP* and *BRACHYURY*. PC-3 (C) and C4-2 (D) cells were transfected with non-specific control (siC) or a pool of *MALAT1*-specific siRNAs. 48 h after transfection, cells were harvested for ChIP assay with non-specific IgG or H3K27me3 antibody. Data are means ± S.D. from experiments with three replicates. *P < 0.01.

Since our data suggest that EZH2 binds directly to *MALAT1* and enhances EZH2-mediated gene repression, we sought to determine whether *MALAT1* promotes EZH2 occupancy and increases H3K27me3 level at EZH2 target loci. Chromatin immunoprecipitation (ChIP) assay using EZH2 and H3K27me3 antibodies demonstrated that *MALAT1* knockdown not only decreased EZH2 recruitment to the promoters of its target genes *DAB2IP* and *BRACHYURY* in PC-3 and C4-2 cells (Figure 4A and 4B), but also decreases H3K27me3 levels at these gene promoters in both cell lines (Figure 4C and 4D). These results indicate that *MALAT1* facilitates EZH2 targeting and enhances the H3K27me3 activity of EZH2 at its target gene loci in CRPC cells.

### *MALAT1* enhances EZH2-mediated PCa cell invasion and migration

**Figure 5:**
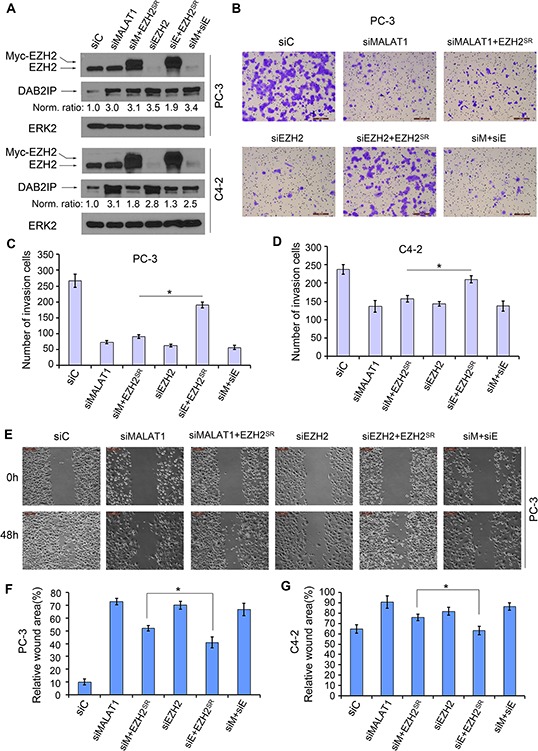
*MALAT1* facilitates EZH2-mediated PCa cell migration and invasion **A.** PC-3 and C4-2 cells were transfected with non-specific control (siC), *MALAT1*- and/or EZH2-specific siRNAs (siM and siE, respectively) in combination with siRNA-resistant EZH2 expression vector (EZH2^SR^). At 48 h after transfection, cells were harvested for western blot analysis using the indicated antibodies. ERK2 was used as a loading control. The western blot density of DAB2IP was first normalized to the density of ERK2 in each lane and then normalized further to the value in cells transfected with siC. **B–D.** Cells were transfected as in (A). At 48 h after transfection, cells were used for Matrigel invasion assays. Representative images of invasion assay performed in PC-3 cells are shown in (B) and the quantification results from PC-3 and C4-2 cells are shown in (C) and (D), respectively. Scale bar, 200 μm. Data are means ± S.D. from experiments with three replicates. *P < 0.01. **E–G.** PC-3 and C4-2 cells were transfected with indicated siRNAs and plasmids as in (A). At 24 h after transfection, artificial wounds were created on cells grown in confluence. Images were taken at 0, 48 h after wound. Representative images from PC-3 cells are shown in (E) and the quantification results are shown in (F) and (G). Scale bar, 50 μm. Data are shown as means ± S.D. from three individual experiments. *P < 0.01.

It has been reported that EZH2 promotes invasion and migration in PCa cells [13, 22, 23]. Since *MALAT1* enhances EZH2-mediated repression of the EZH2 target gene *DAB2IP*, an inhibitor of cell invasion and migration [22, 24], we sought to determine whether *MALAT1* enhances EZH2-promoted invasion and migration in CRPC cells. We demonstrated that knockdown of either *MALAT1* or EZH2 decreased invasion in both PC-3 and C4-2 cells (Figure 5A–5D and Supplementary Figure S1A and S1B). Concomitant knockdown of EZH2 did not further inhibit cell invasion compared to the effect of *MALAT1* or EZH2 knockdown alone (Figure 5A–5D and Supplementary Figure S1A and S1B). Importantly, we demonstrated that EZH2 knockdown-impaired invasion of PC-3 and C4-2 cells was largely reversed by restored expression of siRNA-resistant EZH2 (Figure 5A–5D and Supplementary Figure S1B). In contrast, ectopic expression of EZH2 protein in *MALAT1* knockdown cells had only very minimal rescue effect on cell invasion although the EZH2 protein levels were comparable under these two conditions (Figure 5A–5D and Supplementary Figure S1A and S1B). Similar results were obtained from migration assays in PC-3 and C4-2 cells (Figure 5E–5G and Supplementary Figure S1C). These findings suggest that the effects of *MALAT1* on PCa cell invasion and migration are mediated, at least in part through EZH2. In agreement with these observations, knockdown of *MALAT1* caused derepression of the EZH2 target DAB2IP, an inhibitor of migration and invasion (Figure 5A), but little or no further increase in DAB2IP expression in *MALAT1* and EZH2 co-knockdown cells (Figure 5A). Taking together, we demonstrated a role of *MALAT1* in promoting CRPC cell migration and invasion and this effect appears to be mediated, at least partially through EZH2.

### *MALAT1* promotes expression of PRC2-independent targets of EZH2 in CRPC cells

**Figure 6:**
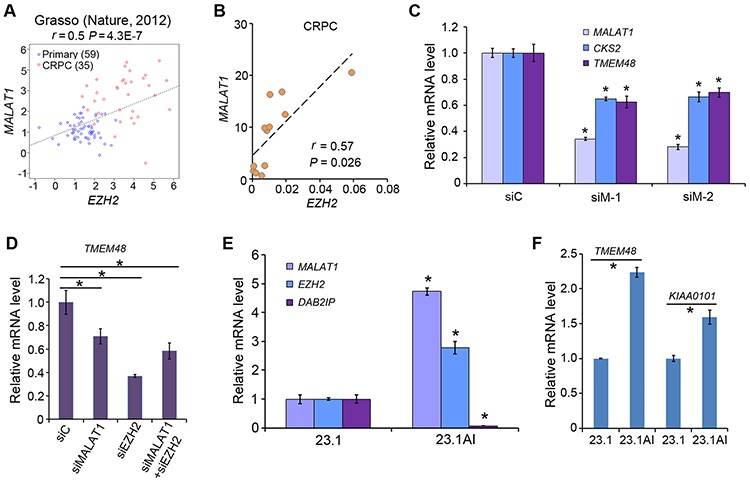
*MALAT1* expression correlates with EZH2 mRNA levels and *MALAT1* enhances expression of PRC2-independent target genes of EZH2 in CRPC cells **A.** Meta-analysis of correlation between *MALAT1* and *EZH2* mRNA expression in human primary and castration-resistant PCa specimens. Pearson correlation test *r* = 0.5, *P* = 4.3E-7. **B.** RT-qPCR analysis of expression of *MALAT1* and *EZH2* mRNA in 12 cases of human CRPC specimens. Pearson correlation test *r* = 0.57, *P* = 0.026. **C.** RT-qPCR analysis of *MALAT1, CKS2* and *TMEM48* in C4-2 cells at 48 h after transfected with control siRNAs (siC) or *MALAT1*-specific siRNAs (siM-1 and siM-2). Data are means ± S.D. from experiments with three replicates. *P < 0.01 comparing to the siC group. **D.** The effect of *MALAT1* on *TMEM48* expression is mediated through EZH2. C4-2 cells were transfected with non-specific control (siC) or *MALAT1*- and/or EZH2-specific siRNA and at 48 h after transfection cells were harvested for RNA isolation and RT-qPCR analysis of expression of *TMEM48*. Data are means ± S.D. from experiments with three replicates. *P < 0.01. **E.** mRNA expression of *MALAT1, EZH2* and *DAB2IP* in androgen-dependent (LuCaP 23.1) and castration-resistant (LuCaP 23.1AI) xenograft tumors analyzed by RT-qPCR. Data are means ± S.D. from experiments with three replicates. *P < 0.01 comparing the data in 23.1AI with those in 23.1 tumors. **F.** RT-qPCR analysis of *TMEM48* and *KIAA0101* expression in LuCaP 23.1 and LuCaP 23.1AI xenograft tumors. Data are means ± S.D. from experiments with three replicates. *P < 0.01.

Both EZH2 and *MALAT1* are highly expressed in human prostate cancers, especially metastatic CRPC [7, 9, 14, 25]. Based on our findings that *MALAT1* binds to EZH2 and enhances EZH2 functions in CRPC cells, it would be clinically significant to determine the correlation between *MALAT1* and EZH2 expression in human CRPC samples. Meta-analysis of a gene expression dataset in public domain [26] revealed that there exists a positive correlation (*r* = 0.5, *P* = 4.3E-7) between *MALAT1* and *EZH2* mRNA expression in human primary and castration-resistant PCa specimens (Figure 6A). The positive correlation between *MALAT1* and *EZH2* mRNA expression was further confirmed by RT-qPCR in an independent cohort of human CRPC samples (*r* = 0.57, *P* = 0.026) (Figure 6B). It has been reported previously that Polycomb (or PRC2)-independent functions of EZH2 are important for castration-resistant progression of PCa [27]. Thus, we sought to determine the role of *MALAT1* in EZH2-mediated expression of Polycomb-independent genes in CRPC cells. To this end, we knocked down *MALAT1* with two individual siRNAs in C4-2 cells and examined expression of *CKS2* and *TMEM48*, two Polycomb-independent genes of EZH2 identified recently [27]. Both *CKS2* and *TMEM48* were significantly downregulated in *MALAT1* knockdown cells (Figure 6C). As expected, knockdown of EZH2 also decreased expression of *TMEM48* (Figure 6D), but concomitant knockdown of EZH2 and *MALAT1* did not further decrease the expression of this gene in C4-2 cells (Figure 6D), suggesting that *MALAT1*-induced expression of Polycomb-independent EZH2 target genes is mediated through EZH2. Moreover, in line with the findings in human CRPC tissues and CRPC cells in culture, we demonstrated that both *MALAT1* and EZH2 mRNAs were expressed at much higher levels in castration-resistant patient-derived xenografts (23.1AI) than in androgen-dependent counterparts (23.1) (Figure 6E). Accordingly, expression of Polycomb-dependent EZH2-repressed gene *DAB2IP* was significantly lower whereas expression of Polycomb-independent EZH2-activated target genes *TMEM48* and *KIAA0101* was significantly higher in 23.1AI xenografts in comparison to 23.1 androgen-dependent xenografts (Figure 6E and 6F). These results indicate that *MALAT1* regulates expression of both Polycomb-dependent and -independent EZH2 target genes and that co-expression of *MALAT1* and EZH2 associates with CRPC progression.

## DISCUSSION

LncRNAs are implicated in regulation of development and tumorigenesis. *MALAT1* is one of the highly cancer-relevant lncRNAs, expression of which is associated with progression of lung, pancreatic, prostate cancer and glioma and therefore, it has been proposed that *MALAT1* could be a biomarker of several cancer types [3–5, 7]. To date, however, the precise mechanism of action of *MALAT1* in oncogenesis is largely unclear. Recent studies indicate that *MALAT1* enhances the activity of the PRC2 complex in bladder cancer by interacting with the subunit SUZ12 but not the subunit EZH2 in PRC2 [18]. Surprisingly, a most recent report shows that *MALAT1* promotes the activation of PRC2 by binding to EZH2 and enhances EZH2-mediated repression of Polycomb-dependent target gene E-Cadherin in clear renal cancer [19]. At present, however, the molecular basis of the interaction between *MALAT1* and EZH2 is unclear. By performing high throughput EZH2 RIP-seq in LNCaP-Rf CRPC cells, we identified *MALAT1* as one of the lncRNAs that binds to EZH2. We not only confirmed their interaction in different CRPC cell lines, but also provided evidence that *MALAT1* interacts directly with EZH2. Importantly, we demonstrated for the first time that the N-terminal of EZH2 and the 3′ end of *MALAT1* are responsible for their interaction. Thus, using various means we have demonstrated the interaction between *MALAT1* and EZH2 in CRPC cells and unravel the mechanism underlying their interaction.

Highly conserved triple helical structures at the 3′end of *MALAT1* was reported to be responsible for the stability and high level expression of *MALAT1* in cells [28]. Moreover, the 3′-end portion of *MALAT1* is not only found highly mutated in colorectal cancer cells, but has also been shown to be essential in various biological processes such as cell proliferation, migration and invasion [29]. Our study demonstrates that the 3′ end of *MALAT1* (nucleotides 7501–8708) preferentially binds to EZH2. These data imply that the structure of the 3′ end of *MALAT1* may also be vital in regulating the oncogenic activity of EZH2 and EZH2-mediated progression of CRPC cells.

EZH2 is the catalytic component of PRC2 complex that is responsible for H3K27me3 and repression of Polycomb-dependent target genes. A recent study identifies a Polycomb-independent oncogenic function of EZH2 in CRPC cells [27]. In the current study we found that *MALAT1* binds to EZH2 and enhances EZH2-mediated repression of Polycomb-dependent target genes such as *DAB2IP* and *BRACHYURY*. Furthermore, we provided evidence that *MALAT1* also enhances Polycomb-independent function of EZH2 and is involved in regulation of expression of EZH2-activated genes such as *CKS2, TMEM48* and *KIAA0101* in CRPC cells. This data indicates that *MALAT1* represents a viable target to inhibit the Polycomb-dependent and -independent oncogenic functions of EZH2 in CRPC. Thus, *MALAT1* regulation of EZH2 may provide new directions for development strategies for treatment of CRPC.

## MATERIALS AND METHODS

### Cell lines and cell culture

C4-2 cells were purchased from UroCorporation. PC-3 and LNCaP cells were purchased from ATCC. LNCaP-Rf, a castration-resistant subline of LNCaP was described previously [21]. LNCaP-Rf and C4-2 cells were cultured in RPMI 1640 medium supplemented with 10% charcoal-stripped fetal bovine serum (FBS) (Life Technologies) (or called androgen-depleted medium) and 100 μg/ml penicillin-streptomycin-glutamine (Life Technologies) at 37°C with 5% CO2. PC-3 and LNCaP cells were cultured in RPMI 1640 medium supplemented with 10% fetal bovine serum (FBS) (Life Technologies).

### Plasmids and antibodies

EZH2 fragments (1–173, 174–335, 336–554, 555–746) and EZH2-N (1–554) were amplified by PCR and subcloned into pGEX-4T-1 vector (GE Healthcare Life Sciences) to generate recombinant GST-EZH2 fusion proteins. Different regions of *MALAT1* (M1-M6) were amplified from purified cDNA of C4-2 cells and subcloned into the backbone vector pcDNA3.1 (Life Technologies). Antibodies used are as follows: anti-EZH2 (XP, Cell Signaling Technology); anti-EED (Millipore); anti-DAB2IP (Abcam); anti-SUZ12 and anti-ERK2 (Santa Cruz Biotechnology); and anti-GST (GE Healthcare Life Sciences).

### Prostate cancer xenografts

The androgen dependent (AD) (LuCaP23.1) and castration-resistant (or androgen independent, AI) (LuCaP23.1AI) xenografts were originally kindly provided by Dr. Robert L. Vessella (Department of Urology, University of Washington Medical Center, Seattle, WA). AD LuCaP xenografts were propagated in BALB/c nu/nu mice and AI xenografts were propagated in SCID mice as described previously [30].

### Native RNA immunoprecipitation-coupled high throughput sequencing (RIP-Seq)

LNCaP-Rf cells grown to ~ 70–80% confluence were washed twice with 1 × PBS and trypsinized. After washing with ice-cold 1 × PBS, cell lysate was obtained by centrifugation (12,000 rpm for 10 min at 4°C) after incubation with RIP buffer [50 mM Tris-HCl (pH 7.9), 0.25 M NaCl, 1% Nonidet P-40 (NP-40) 10 mM EDTA and RNase inhibitor (Promega)] for 30 min. Cell lysate was incubated with antibody/beads for 18 h at 4°C. Protein-RNA complexes bound to beads were washed three times in NT2 buffer [50 mM Tris-HCl (pH 7.4), 300 mM NaCl, 1 mM MgCl2, 0.05% Nonidet P-40 (NP40),1 × PIC, RNase inhibitor], followed by treated with DNase I for 15 min at 37°C and further washed twice with NT2 buffer. Co-purified RNA was extracted by RNA purification kit (RNAeasy Mini Elute kit, QIAGEN) and cDNA was synthesized using the SuperScript kit from Life Technologies and the quality of cDNA was analyzed by real-time polymerase chain reaction (PCR). Double-strained cDNA was synthesized and fragmented for next generation sequencing. RIP-seq raw reads were mapped to human reference genome (hg19/GRCh37) using Tophat (v1.4.0) [31]. Raw count mapped to each Refseq gene was calculated using RSeQC package [32]. Hypergeometric test was applied to evaluate the significance of enrichment of RIP-seq reads at each gene.

### RNA interference

Smart pool of siRNAs targeting EZH2 and *MALAT1* and nonspecific control siRNAs were purchased from Dharmacon. Two individual siRNA targeting human *MALAT1* (siM-1 and siM-2) were synthesized by Dharmacon. siRNA transfection of cells was performed following the manufacturer's instruction.

### RT-qPCR

Total RNA was isolated from cells with TRIzol and cDNA was synthesized using the RT-PCR kit from Promega. Three-step real-time polymerase chain reaction (PCR) was performed using the SYBR Green Mix (BioRad) and an iCycler Iqtm system (BioRad). The primer sequences used for PCR are described in Supplementary Table S1.

### Chromatin immunoprecipitation (ChIP) assay

ChIP assay was performed as described previously [33]. Briefly, cells were crosslinked with 1% formaldehyde at room temperature. After washing with 1 × PBS, cell nuclei were extracted with lysis buffer 1 (50 mM HEPES, pH 7.5, 140 mM NaCl, 1 mM EDTA, 10% glycerol, 0.5% Nonidet P-40, 0.25% Triton X-100) and washed with lysis buffer 2 (10 mM Tris-HCl, pH 8.0, 200 mM NaCl, 1 mM EDTA). Cell nuclei was resuspended in lysis buffer 3 (10 mM Tris-HCl, pH 8.0, 100 mM NaCl, 1 mM EDTA, 0.1% sodium deoxycholate) and sonicated. Cell lysis was incubated overnight at 4°C with 10 μg antibody bound-protein G beads (Life Technologies). After several time washing, DNA-protein complex was elucidated, reverse crosslinked and purified. Real-time PCR was used to detect the amount of DNA immunoprecipitated by antibody.

### Wound healing assay

C4-2 cells were transfected with indicated siRNA and seeded into six-well plates. Artificial wounds were created on the cell monolayer using culture-inserts for live cells analysis. Migrated cells and wound healing were visualized at 0 and 48 h. For each group, at least 3 artificial wounds were photographed immediately and at the time points indicated after the wound formation. Cell migration was evaluated by measuring the difference of wound areas.

### Invasion assay

*In vitro* invasion assay was performed using BioCoat Matrigel invasion chamber (BD Biosciences) according to the manufacturer's protocol. PC-3 and C4-2 cells were transfected with indicated siRNAs for 24 h and cultured in the insert for 24 h. Cells were fixed in methanol for 15 min and then stained with 1 mg/ml crystal violet staining for 20 min. At least 5 fields for each group were photographed after staining. Invasion was evaluated by counting the number of the invaded cells.

### Prostate cancer patient samples for RT-qPCR analysis

RT-qPCR analyses of *MALAT1* and EZH2 mRNA expression in CRPC patient samples were approved by the Mayo Clinic Institutional Review Board (IRB). CRPC patient samples were selected randomly from patients who have been treated at Mayo Clinic between January 1995 and January 2014. The age of the patients ranged from 45 to 78 years. Total RNA was isolated from 10 micron section of FFPE samples using the Ambio RNA Isolation kit (Thermo Fisher Scientific) with DNase treatment and RT-qPCR was performed to detect *MALAT1* and EZH2 expression.

### Statistics

Experiments were carried out with three or more replicates. Statistical analyses were performed by two-tailed Student's *t* test. *P* < 0.05 is considered statistically significant.

## SUPPLEMENTARY FIGURE AND TABLE


