# Supplementary material for: LncRNA MALAT1 enhances oncogenic activities of EZH2 in castration-resistant prostate cancer
Source: Oncotarget. 2015 Oct 15;6(38):41045–55. doi: 10.18632/oncotarget.5728 (PMC4747388; doi:10.18632/oncotarget.5728)
Supplement: Supplementary file 1 [file oncotarget-06-41045-s001.pdf]

SUPPLEMENTARY FIGURE AND TABLE

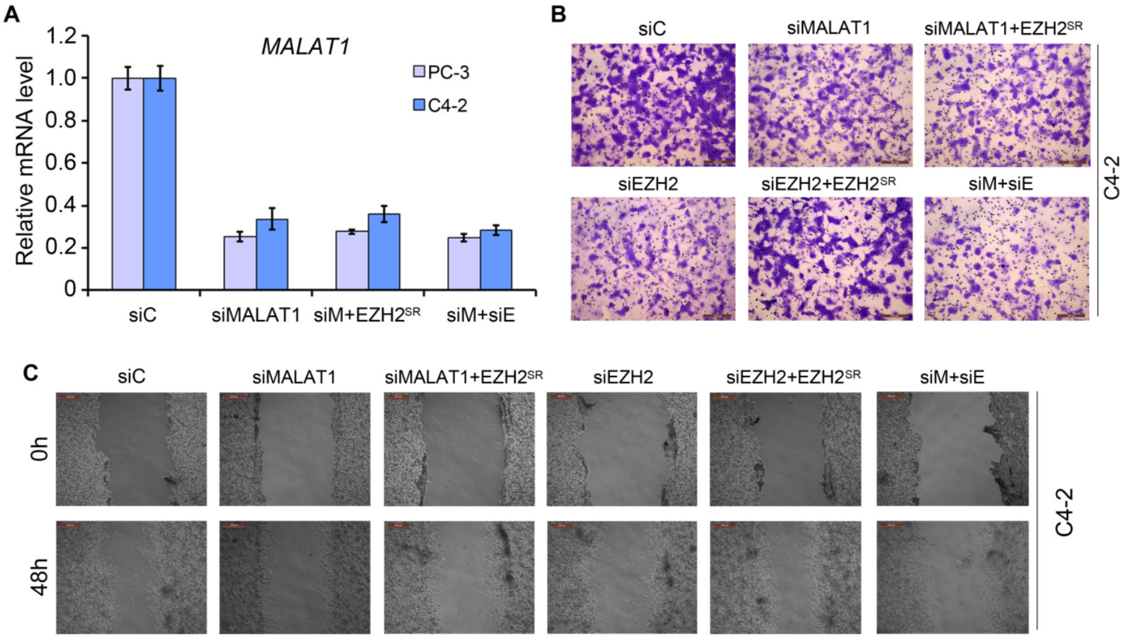

Supplementary Figure S1: *MALAT1* facilitates EZH2-mediated PCa cell migration and invasion. PC-3 and C4–2 cells were transfected with siRNAs and/or siRNA-resistant EZH2 expression vector (Myc-EZH2^{SR}) as indicated in Figure 5A. **A.** At 48 h after transfection, RT-qPCR analysis of *MALAT1* expression in PC-3 and C4–2 cells transfected with control siRNA (siC) and *MALAT1*-specific siRNA (siMALAT1 or siM). Data are means ± S.D. from experiments with three replicates. **B.** C4–2 cells were transfected as indicated in Figure 5A. At 48 h after transfection, cells were used for matrigel invasion assays. Representative images of invasion assay are shown. **C.** At 24 h after transfection, artificial wounds were created on C4–2 cells grown in confluence. Images were taken at 0, 48 h after wound. Scale bar, 200 μm.

Supplementary Table S1: Information of the primers, sequences of siRNAs/shRNAs used

MALAT1 Cloning Primers

M1-F	CCCAAGCTTGGGGTAAAGGACTGGGGCCCCGCAACT
M1-R	TATGAAGACTTAGAAGAGTACCGCTCGAGCGG
M2-F	CCCAAGCTTGGGGCATGAGGAAGGAAAAGATA
M2-R	ACCACTCGCTTTCCCTGTCCGCTCGAGCGG
M3-F	CCCAAGCTTGGGTGGTAAAAATCCGTGAGGTC
M3-R	CACTACCATATCCAAACAACCCGCTCGAGCGG
M4-F	CCCAAGCTTGGGTGTGGTTCTCTTTTGGAATT
M4-R	TGGTCCATTAAAGAGTGTTCCCGCTCGAGCGG
M5-F	CCCAAGCTTGGGGATCAGGATTTGAGCGGAAG
M5-R	TTGTTGTCTCTCCTGCCACACCGCTCGAGCGG
M6-F	CCCAAGCTTGGGAGCGCTATTATCCTAAGGTC
M6-R	TTTAGAGCTTCTCCATTTCCGCTCGAGCGG

(*Continued*)

MALAT1 Cloning Primers

**RT-qPCR primers**	
DAB2IP-F	ACACGCCATGGAGCCCGACT
DAB2IP-R	GAAGCCCGTGACCCGGAACG
BARCHYURY-F	AGGTGGGGAAGTTTCCTTCT
BARCHYURY-R	GCAAATGAGGTCCTTTTGGT
M1-RT-F	TTGAGGCGTTTTCCAAGAGT
TMEM48-RT-F	AGGTCGCGGGACATACTGT
TMEM48-RT-R	TGCAGATGGGTAGAAATAGCACT
CKS2-RT-F	TTCGACGAACACTACGAGTACC
CKS2-RT-R	GGACACCAAGTCTCCTCCAC
KIAA0101-RT-F	ATGGTGCGGACTAAAGCAGAC
KIAA0101-RT-R	CCTCGATGAAACTGATGTCGAAT
M1-RT-R	CGGTTGAGAAGTGGCAAAAT
M2-RT-F	GACGGAGGTTGAGATGAAGC
M2-RT-R	ATTCGGGGCTCTGTAGTCCT
M3-RT-F	CCCACCCCCTTAATCAGACT
M3-RT-R	CAACAGCACAGCGGTACACT
M4-RT-F	GTGTGCCAATGTTTCGTTTG
M4-RT-R	AGGAGAAAGTGCCATGGTTG
M5-RT-F	AAAGCAAGGTCTCCCCACAAG
M5-RT-R	GGTCTGTGCTAGATCAAAAGGCA
M6-RT-F	CTCCCCACAAGCAACTTCTC
M6-RT-R	TTCAACCCACCAAAGACCTC
FOXO1-RT-F	CGTGCTTACAGCCTTCTA
FOXO1-RT-R	ACCTCCATCGTGACAAAA
**ChIP-qPCR primers**	
DAB2IP-F	CCTGCTTTCTGTTTCCTTCTCCTG
DAB2IP-R	TTGAACCACCTCCTCCTCCCTCTC
BARCHYURY-F	AGGTGGGGAAGTTTCCTTCT
BARCHYURY-R	GCAAATGAGGTCCTTTTGGT
**siRNA sequences**	
non-specific siRNA/shRNA	UAGCGACUAAACACAUCAA
siEZH2 (SMART pool)	CAAAGAAUCUAGCAUCAUA
	GAGGACGGCUUCCCAAUAA
	GCUGAAGCCUCAAUGUUUA
	GAAUGGAAACAGCGAAGGA
MALAT1-siRNA (siM1)	GACCUUGAAAUCCAUGACGUU
MALAT1-siRNA (siM2)	GAUCCAUAAUCGGUUUCAAGGUU
